# Cesarean Section on a Child Twelve Years and Eight Months Old, with Contracted Pelvis, for Severe Puerperal Eclampsia

**Published:** 1906-04

**Authors:** H. J. Boldt

**Affiliations:** New York


### SPECIAL SELECTION

### Cesarean Section on a Child Twelve Years and Eight Months Old, with Contracted Pelvis, for Severe Puerperal Eclampsia.

By H. J. BOLDT. M. D., New York.

*(Post-Graduate, December, 1905.)*

SINCE Halbertsma, of Utrecht, in 1889, advocated for the first time in the medical press the operation of Cesarean section for puerperal convulsions, the number of cases has multiplied to such an extent that a review of them will cause an unbiased critic to form the opinion that such operation is indicated in certain selected cases. Hillman[1] tabulated 40 cases, in which 21 mothers died and 19 recovered; of the 41 children, 18 died and 23 remained alive. Seven times the convulsions continued after the Cesarean section.

Olshausen[2] at the meeting of the Berlin Gynecological Society of November 24, 1899, presented a woman upon whom he had performed a classical Cesarean section for eclampsia; the fate of the child is not stated. He said that with an intact cervix and an undiluted os classical Cesarean section was indicated. If, however, the cervix was dilated, a vaginal accouchement was preferable.

Sippel[3] did the operation on a 17-year-old primipara five weeks before the normal termination of pregnancy, and the mother died on the seventh day, of hemorrhage from a duodenal ulcer which, however, had no influence on the satisfactory result brought about by the operation. The child lived.

Koetschau[4], at the meeting of the Cologne Gynecological Society, referred to two cases in which he had operated in 1898 both women recovering, although one had subsequently died of pneumonia. One child lived; the other had succumbed before the section was done. He said that the heroism did not lie in the performance of the operation, but it was heroic to omit it when the conditions present plainly indicated its performance.

Ribbius[5] at a meeting of the Netherlands Gynecological Society, narrated a case of Cesarean section with recovery. Salin reports one case, the patient dying of septic endocarditis caused by infection through pneumonia acquired by the inspiration of foreign material during the convulsions. Hans Lowenstein[7] reports three cases of the severest form of eclampsia; two of the children survived, but all of the mothers died.

Leopold Prinz[8] has tabulated 55 cases of Cesarean section for eclampsia, which gave a modified mortality of 42.42 per cent. The infantile mortality, if the deaths following during the first days after delivery are added, was 55.8 per cent. E. Streckeisen[9] reports two cases. In the first, the woman died of peritonitis on the third day. The section brought forth two dead, macerated children. In the second case the woman recovered; both children, also twins, lived, although one died from convulsions six weeks after its birth.

Halliday Croom[10] reports two cases, in both of which the mother died; one from convulsions, the other of pneumonia. Wanner[11] reports one case of operation when the mother was in a moribund condition. She died but the child remained alive.

My own case is the following: E. H., aged twelve years and eight months. She was seen by me when about five months pregnant (no exact dates could be elicited). There was a generally contracted pelvis. The distance from the lower border of the symphysis to the promontory of the sacrum (finger measurement), was two and a half inches; from the lower border of the symphysis to the tip of the coccyx, two and a quarter inches. The transverse diameter was approximately two and a half inches. The question of producing an abortion arose, but after consideration the proposal was declined. The pregnancy was thought to be at term between the 15th and the 20th of August.

On August 9, 1905, the child was admitted to my service at the Post-Graduate Hospital, it being my intention to do an elective Cesarean section before the class on August 12th. The urine showed a trace of albumin and a few hyalin casts. There were no symptoms present to cause one to apprehend danger, except the very slight trace of albumin in the urine. On the evening of August 10, without any prodromes, the patient was suddenly seized with a very severe eclamptic attack, for which a hypodermic injection of one-third of a grain of morphin, with other appropriate treatment was used. I was communicated with at once by telephone, and saw the girl within an hour. In that interval, however, she had had five additional convulsions. She was unconscious on my arrival, and had been in that condition from the time of the first convulsion. The cervix was well formed and neither dilated nor dilatable. The heart sounds of the child were at that time audible but weak.

While I was considering which procedure would be best to follow in the interest of the patient another very severe convulsion occurred. With this she began to get pulmonary edema, which increased in intensity from minute to minute. The face was livid, the pulse small and very rapid. The administration of oxygen was at once begun and the patient ordered to the operating room to be delivered by the Cesarean section. Her condition was, however, such that it was thought doubtful if she would live long enough to be delivered. Within an hour and a half after the first convulsion she was delivered of her child by Cesarean section. The uterus extended up to the diaphragm, pushing the other abdominal viscera aside. The child was in the L. O. P. position and was extracted with the placenta in exactly two minutes after the beginning of the section, and in 29 minutes after the beginning of the operation the patient was returned to bed. This is mentioned only to show that no time was lost.

The paient began to improve very perceptibly from the moment the uterus was emptied, so that we had reason to hope recovery might take place if the convulsions did not recur. The improvement was especially noticeable in the rapid subsidence of the pulmonary edema, which had been very alarming, the frothy mucus coming from the mouth and nose in a *continuous stream.* An intravenous infusion of 1500 c.c. of saline solution had been given, and the hot pack used. No convulsions took place until after midnight; then, however, one occurred, and from that time on they recurred at comparatively short intervals despite all therapeutic measures. At about 9 A. M. pulmonary edema also recurred and the little patient died at noon. The child is living.

*Autopsy* by Professor Brooks, three hours after death. A large quantity of fluid was found in the pericardium; the right ventricle was firmly contracted, the myocardium degenerated. Lungs edematous; pleuritic adhesions on the right side. Liver: numerous echymoses on Glisson's capsule; fatty degeneration of the liver; a large nodule, three centimeters in diameter, yellowish white in color and of firm resistance, near the surface of the organ. The kidneys were typical of very acute parenchymatous nephritis and beginning fatty degeneration. Both ureters were dilated to three times their normal caliber, especially above the brim of the pelvis.

The urine, another specimen having been taken after the first convulsion, was solid from the presence of albumin, and it was crowded with casts. That there was an absolute indication for the therapeutic procedure adopted in this case no one can doubt. The same may be said of all the cases reported in literature. They were apparently all hopeless cases without operation. We cannot consider the operation as a certain means of saving either mother or child, but must look upon it as one of the most energetic and sometimes successful attempts to save life when other therapeutic measures fail. In the case here related I have the satisfaction of having at least saved the life of the child, which is still living and doing well.

There is no unanimity of opinion as to the treatment of puerperal convulsions, especially as to when Cesarean section is indicated. It is therefore important that when the operation is performed for the relief of this dreaded ailment, the operator should state the reasons precisely which induced him to formulate the indication. The unanimous concensus of opinion, however, is that it is desirable to empty the uterus as soon as it can be done, with as little traumatism as possible, because experience has taught us that in the greater number of instances the convulsions cease entirely or become much milder after the uterus has been emptied of its contents. This shows that in at least some cases there must be a mechanical obstruction to the elimination of the poison which causes the convulsions; this was also shown in this case by the dilatation of the ureters, a condition which has also frequently been present in other cases that have been reported.

All the ascribed causes so far known for eclampsia must to a large extent be considered as theoretical. We can best explain the cause of the disease under the somewhat crude term of autointoxication, because we cannot find any external cause for the poisonous toxins which produce the convulsions and fever, and cause such detrimental effects on the liver and kidneys. That there are other causes than mechanical obstruction to the elimination of the toxic poison is shown by the continuance of the convulsions after delivery in some cases, and the fact that sometimes puerperal convulsions do not occur at all until after delivery in other cases. Nevertheless, when the symptoms are urgent in an undelivered woman, delivery should be accomplished at the earliest possible moment by whichever method seems most promising to mother and child. It is not good practice to delay until extreme symptoms have made headway, when, in addition to pulmonary edema, cardiac paralysis and hemorrhages into the brain and other organs may take place. We also have the danger of secondary pneumonia from aspiration of foreign substances.

The indication for a classical Cesarean section of course occurs very seldom, but when it does occur, it should not be delayed until the patient is in *extremis* if it can be sooner done. I should formulate the indication, if the symptoms were so extreme that the life of the mother and child were in danger, and when the cervix was still neither dilated nor dilatable, so that delivery could not be accomplished by way of the natural passages, when even the vaginal Cesarean section according to the method of Duehrssen would involve greater risk than the classical Cesarean section, in the hands of a competent operator.

In connection with the case reported there are some interesting legal features. To one not acquainted with legal matters it does not seem that there is anything about the case requiring the attention of the authorities, yet when the death certificate was presented to the Board of Health, it was declined and a coroner's investigation ordered. On the arrival of that official he severely commented on the fact that the operation had been performed without first notifying the coroner's office that such an operation had been contemplated, because the patient was a minor. Further, he condemned the performance of an autopsy on the body. He maintained that the coroner's office should have been notified of the admission of the patient to the hospital.

From the view of a medical man, I cannot even now see that an unlawful act has been committed by the hospital authorities or myself. To notify the authorities as soon as the patient came under observation would have been a violation of professional confidence, and in my opinion would have been a valid reason for a civil suit against the informer. To request the coroner to come for the purpose of taking an antemortem statement from the patient before performing the operation, would have been unjustifiable, even if the patient had been conscious, because it would have had a very detrimental mental effect. Neither can I see that an infringement of the law was committed in having an autopsy performed so long as the written consent thereto had been obtained from the mother of the patient. It was the duty of the coroner to find out, if possible, who the person was who was responsible for the pregnancy, but under the circumstances the attempt was not justifiable, so far as the coroner was concerned, until it was too late.

1. *Monatsschrift für Geburtshülfe und Gynakologie,* Vol. X, p. 193.

2. *Ibid.*, Vol. XI, p. 602.

3. *Ibid.*, Vol. XIV, p. 280.

4. *Ibid.*, Vol. XV, p. 233.

5. *Ibid.*, Vol. XVI, p. 115.

6. *Ibid.*, Vol. XVII, p. 1373.

7. *Centralblatt für Gynakologie*, 1902, p. 117.

8. *Ibid.*, 1904, p. 122 (abstracted).

9. *Ibid.*, 1903, p. 808 (abstracted, Inaug.-Diss., Zurich, 1903).

10. *Ibid.*, 1904, p. 1292; *Lancet*, June 18, 1904.

11. *Ibid.*, 1904, p. 1339.

